# "Is Every Woman a Nurse"?

**Published:** 1918-11-16

**Authors:** 


*To the Editor of* THE HOSPITAL.

### "Is Every Woman a Nurse"?

*To the Editor of* THE HOSPITAL.

SIR,—"Latent or developed the call is there." How can the matrons of training-schools criticise the above sentences? From their standpoint they could truthfully answer that every woman is not a nurse. A true nurse must be born. A nurse can be trained to be a mechanical worker. She has to make it commercial, because the working woman's labour is her capital. There are few women with incomes that take up the profession for the pure love of nursing at the present time. I am not speaking of military nursing. Women have to start life and earn their living; they want a respectable profession, and many of them a home; and the nursing profession presents to the applicants what they seek. There has not always been a shortage of nurses, and it has been my experience to meet some fine characters as colleagues, and to give a helping hand to many, some of whom have sent me their thanks for my interest when they have succeeded in obtaining their certificate in general hospitals. Why does "Ierne" waste time sowing seed of disloyalty, grumblings, and discontent? Any right-minded matron knows that reform is needed in many things, and she would make many concessions and alter many things were she in a position to do so. Committees appoint matrons. They do not fix the salaries of their staff, and those that pay the piper should have a choice in calling the tune. So my advice to "Ierne" is, Don't attack the matrons but attack the committees, if an attack must be made, to obtain a reform in nurses' conditions of labour, pay, living, and off-duty time. Sixty years ago is a long time to look back, and the committees, governors, superintendents, and matrons of to-day see a great change in all things appertaining to hospital life—applicants, conditions, appliances, and methods of training nurses. When Walter Scott wrote the words "Ierne" quoted,

"When pain and anguish rend the brow,
A ministering angel thou,"

he meant the context to be read, and I vow that Marmion would not call every woman that is born a ministering angel, for we must take all collectively. The man that makes character makes foes, and so it is with many matrons; they try to make character with material that does not always make good weft and warp, and in the making of this material they learn that every woman is not a nurse, and though she may learn the theoretical methods of nursing she has not

"The reason firm, the temperate will,
Endurance, foresight, strength and skill.
A perfect woman nobly planned,
To warn, to comfort and command,
And yet a spirit still and bright,
With something of angelic light."

Has "Ierne" adopted Jack London's plan when he wrote "The People in the Abyss"; lived in the hospitals, seen the methods, life, conditions of living; in fact, had all the nurse's experiences, and is not talking from hearsay? A little learning is a dangerous thing, and if we do not possess full knowledge and experience, and if we do not attack the proper persons for a general reform of conditions in hospital, no material good is done by a lot of talk and agitation, and the good result necessary to good fellowship between committees and the nursing staff is succeeded by an undercurrent of bad feeling, dissension, and discontent. Reform in hospital life is moving, but let it begin at the proper source, and it will move surely and swiftly. Let us study all sides, and then decide who are the defaulters and what are the best methods to be used to bring about the best results for the worker as well as for those who control.—Yours faithfully,

LIVERPOOL.

